# Time for change: Overcoming perpetual feelings of inadequacy and silenced struggles in medicine

**DOI:** 10.1111/medu.14030

**Published:** 2019-12-22

**Authors:** Anique Atherley, Stephanie N E Meeuwissen

**Affiliations:** ^1^ Department of Educational Development and Research School of Health Professions Education Maastricht University Maastricht the Netherlands; ^2^ Medical Education Unit School of Medicine Western Sydney University Sydney New South Wales Australia

## Abstract

Focusing on support of psychological wellness in medical students, residents and physicians, this commentary highlights the need for cultural change and the accompanying importance of leadership.

In this issue of *Medical Education*, Gottlieb and colleagues review and summarise the literature on imposter syndrome.^1^ Imposter syndrome describes ʻa pattern of behaviour wherein people (even those with adequate external evidence of success) doubt their abilities and have a persistent fear of being exposed as a fraud.’^2^ The authors uncover increasing evidence of imposter syndrome during both training and medical practice.^1^ This is important because, in an unsupportive culture, imposter syndrome can be associated with constructs related to mental health problems, including pervasive self‐doubt,^3^ anxiety, burnout, depression and suicide.^1^ This, in turn, has implications for health care because these manifestations have been associated with decreased empathy towards patients,^4^ increased substance use^5^ and increased frequencies of medical error.^4^

… in an unsupportive culture, imposter syndrome can be associated with constructs related to mental health problems …

Our medical students, residents and physicians are experiencing high prevalences of anxiety,^6^ experiences of shame,^7^ burnout,^4^, ^5^, ^8^ depression,^9^ suicidal ideation^9^ and, evidently, imposter syndrome.^1^ Gottlieb's et al's review offers yet another signal that it is time for change. In this commentary, we highlight a need for cultural change while stressing how leadership can be key to the optimisation of well‐being for trainees and physicians.

Trainee and physician distress can be influenced by factors operating at four levels that are, respectively, individual, work unit‐related, organisational and national.^10^ Personality traits^1^, ^4^ and female gender^2^, ^3^ are examples of individual‐level factors that make trainees and physicians prone to developing distress. Work unit factors include call schedules, norms and expectations in medical environments, work unit leader behaviours and team structure.^10^ At the organisational level, in education settings, the curriculum has been recognised to affect well‐being.^11^ In the workplace, electronic medical records, culture and work policies are contributory.^10^ Lastly, at a national level, the evolving supervisory roles of physicians, regulations and requirements is impactful.^10^ Despite the benefits shown by individual‐, group‐ and curriculum‐level strategies (e.g. mentorship, workshops and a reduced emphasis on grades),^11^ worrying statistics remain the norm.

Distress is increasingly recognised as an issue of culture in medicine.^12^ Importantly, we must be careful about stigmatising trainees and physicians who are struggling with forms of distress.^13^ In any case, interventions that focus specifically on personal well‐being are unlikely to be sufficient for long‐term improvement if trainees and physicians learn or work in pervasively toxic cultures within suboptimal health care systems. As such, it is important to heed recent articles that stress the need for system‐level changes in culture.^1^, ^3^, ^4^, ^14^

Shanafelt and colleagues wrote: ʻCulture refers to the shared and fundamental beliefs, normative values, and related social practices of a group that are so widely accepted that they are implicit and no longer scrutinised.’^12^ Culture is passed from one generation of doctors to the next.^12^ Traditionally and currently, the learning and workplace culture in medicine is typically one of low psychological safety, silence and feelings of inadequacy. This is evidenced when some supervisors are dismissive of the mistreatment of junior staff, uttering comments such as: ʻBack when I was training, it was done to us too and we are still standing.’ In medical learning and workplace culture, the acts of sharing doubts or seeking help are often perceived as signs of weakness^3^, ^15^ and the perceived hierarchy makes trainees and physicians reticent to speak up about medical errors^15^, ^16^, ^17^ or pursue organisational improvement.^18^ As a result, low psychological safety ultimately has a negative impact on patient care.^16^

In a culture that punishes mistakes and doubts, and promotes silence and performance, it is unsurprising that many trainees and physicians fake competence in order to be accepted.^3^ Additionally, in a system that incorporates a rapid growth in medical knowledge and an increasingly litigious and digital medical practice environment, feelings of inadequacy and self‐doubt are likely to be exacerbated. Systemic, frequent transitions in training contexts may also limit ʻvoice behaviour’ (speaking up),^18^ and precipitate self‐doubt and fear of being exposed as a fraud.^3^ Failing to work towards change in learning and workplace culture in medicine will continue to fuel self‐doubt and fears. In the current culture, many will remain silent and many will continue to feel inadequate. As a result, we should anticipate further increases in distress levels.

In medical learning and workplace culture, the acts of sharing doubts or seeking help are often perceived as signs of weakness …

Change will not come quickly because influencing culture is complex and time‐consuming. How then might we begin to move forward in this regard? We must stress that it is imperative that any intervention is *not* a box‐checking exercise with the superficial creation of ʻpsychological wellness curricula’ simply to satisfy accreditation requirements. Rather, we need the field to *actualise* a nurturing and safe learning and workplace culture that moves towards normalising speaking up and sharing experiences of shame,^7^ self‐doubt, fears of personal inadequacy and making errors throughout training and practice. How do we do that? How do we effect such change? What are the implications for our practice and research agendas?

We specifically challenge institutes to consider their leaders and the trickle‐down effects that leaders can have on the psychological wellness of all

One big piece of the puzzle that has been highlighted specifically in relation to imposter syndrome^2^ is leadership.^10^ Our leaders must believe change is necessary^12^ and these leaders must be drawn from many stakeholder groups including medical students, residents, physicians, educators, curriculum leaders, hospital leaders and policymakers. Leaders in medical education and workplace settings have often been chosen based on scholarly or patient care achievements and rarely on their leadership abilities or their visions for the units and people within their organisations. We specifically challenge institutes to consider their leaders and the trickle‐down effects that leaders can have on the psychological wellness of all. The encouragement of more inclusive, engaging leaders who invite and appreciate input from individuals and units should be the goal. Inclusive leadership improves psychological safety and thus the learning and work climate.^16^, ^17^ Leader training to model inclusive behaviours that flatten hierarchies is paramount.^16^ Leaders themselves should be open about their experiences with self‐doubts and fears and embrace their own vulnerabilities. In so doing, a nurturing and even contagious, safe learning culture may emerge, one in which people feel safe to speak up, in which more dialogues take place about success, uncertainties, shame, fears and errors, and in which trainees and physicians work with compassion and empathy towards their patients, their colleagues and themselves.

Leaders themselves should be open about their experiences with self‐doubts and fears and embrace their own vulnerabilities

This perhaps visionary view first demands that our research efforts better address a number of questions regarding how we might best move forward: How can we train and support leaders to model inclusive behaviours and share stories about personal success, doubts, shame, fears and errors? Where is the path on which increasing feelings of psychological safety might influence *culture* to the point of translation across varied work and learning environments? One thing we cannot continue to question is whether or not change is required. All stakeholders must work together to move beyond holding workshops aimed at making individuals more resilient and, towards building an intentional and proactive culture that is psychologically safe for learning and work; towards an environment that challenges institutional norms and accepts and analyses errors with a view to ensuring their prevention and learning, and towards a system that selects and trains leaders who set good examples and create policies for healthy work and education. The need for change is not hypothetical because negative outcomes continue to exist.^12^ We cannot understate how difficult this will be given that culture is complex^12^ and is dependent on good leaders^12^ and adequate resources. Change will take time, but it is time for change.

In a safe learning culture, people feel safe to speak up, dialogues take place about success, uncertainties, shame, fears and errors, and trainees and physicians work with compassion and empathy towards their patients, their colleagues and themselves
